# On Feigned and Factitious Diseases, Chiefly of Soldiers and Seamen; on the Means Used to Simulate or Produce Them; and on the Best Modes of Discovering Impostors

**Published:** 1844-07

**Authors:** 


### ART. IX.

1. *On Feigned and Factitious Diseases, chiefly of Soldiers and Seamen; on the means used to simulate or produce them; and on the best modes of discovering Impostors.* By HECTOR GAVIN, M.D.—*London*, 1843. 8vo, pp. 436.
2. *On the Enlisting, Discharging, and Pensioning of Soldiers, with the official documents on these branches of military duty.* By HENRY MARSHALL, F.R.S.E., Deputy-Inspector-General of Army Hospitals. Second Edition.—*Edinburgh*, 1839. 8vo, pp. 260.
3. *Memorial de l'Expert dans la Visite sanitaire des Hommes de Guerre, ou examen des principales questions relatives aux maladies et infirmités qui peuvent donner lieu à l'exemption et à la réforme du service de l'armée de terre, et à leur simulation, provocation, et dissimulation.* Par L. FALLOT, D.M., Médecin principal de l'Armée, etc.—*Bruxelles*, 1837. 8vo, pp. 414.

ALTHOUGH feigned diseases exist, to a certain extent, in all classes of society,—largely in workhouses and prisons, and especially in the navy and army—we shall, on the present occasion, confine our observations to the soldiers of the British army. In most particulars our remarks will apply to other departments, or they can be easily made to apply to them by practical men.

The study of feigned diseases is of great importance to the army or navy medical officer, who, in the course of his duty, is called upon not merely to treat disease where it really exists, but frequently to decide whether or not it exists at all. This circumstance renders his position one of considerable difficulty, because he is deprived of a most important aid to diagnosis, being compelled at all times to receive the statement of his patients with caution, and to trust very much to the information afforded by physical signs. As there are few men who will feign disease for the mere pleasure of deceiving, it seems necessary to examine what are the objects to be gained by successful simulation, in order to appreciate the extent to which it is likely to be carried among soldiers and sailors, and the means best adapted for its detection and prevention.

In the British army a recruit enlists of his own free will, but frequently he has no sooner done so than he repents, and endeavours, by false representations, to induce the surgeon before whom he is brought for approval to reject him. Failing in this, or if repentance does not so immediately follow, he commences a system of *malingering* (as it is termed,) on joining at head-quarters, by professing inability to learn some part of his drill, owing to the effects of an injury stated to have been received prior to enlistment, and the traces of which are either very slight or cannot be seen at all; or he throws himself upon the hospital on some pretence or other, and remains there with a perseverance worthy of a better cause, until he either obtains his discharge or, losing all hope of success, returns to his drill, and endeavours to make the best of his bargain. Again, after a soldier has been some time in the service, he becomes wearied with the constant repetition of the same duties, drills, and punishments, and is anxious, at all hazards, to return to civil life. The very nature of military service engenders a love of change; for the soldier being frequently moved from one barrack to another, and seldom occupying any station sufficiently long to attach to it the idea of a home, becomes fond of the excitement of a migratory life. The same feeling by degrees extends to his duties and occupations, with the monotony of which he gets disgusted. The only means of procuring a change is by his escape from the army; but, as he has engaged to serve "until he shall be legally discharged,"—and there is no prospect of this till such time as, from age, infirmity, or disease, he is deemed incapable, by the military authorities, of rendering efficient services—he frequently endeavours, by simulating a disability, to procure a termination of that life-servitude which has now become irksome to him. The Duke of Wellington has stated that "the objection to entering into the army is the severity and regularity of the duty; the regularity of the discipline; and the life which the soldier is obliged to lead, and which you must oblige him to lead; the climates to which he is exposed, and the constancy of the service in those climates."* These objections to entering the service well explain the motives which induce many soldiers, already in it, to endeavour to work out their discharge, after having experienced its inconveniences, and had the golden dreams of the recruiting-serjeants dissipated by sad and disagreeable reality.

Another class of malingerers are those who feign disease to avoid some particular duty, or to evade punishment. Although their object is merely temporary, and does not involve their ultimate loss to the service, it is of considerable importance to detect and frustrate their schemes: in the one case to prevent an act of injustice to the other soldiers; in the other to maintain the discipline of the service.

No soldier can claim his discharge as *a matter of right*, however long he may have been in the army; but if he is discharged in consequence of being unfit for military duty, a pension is granted him as a reward for past services, or as a compensation for the disability under which he labours. Formerly pensions for life were awarded to all men thus discharged, however short a time they might have served; and as they thus secured an annuity at the same time that they obtained their release from the thraldom of military discipline, a powerful inducement existed to feign disease. By recent regulations, however, those only are entitled to permanent pensions who have completed twenty-one years' service;

* Gurwood's Despatches.

while men discharged at an earlier period receive a temporary allowance, proportioned to the length of time they have served, or the nature of the disability for which they have been invalided. As the soldier, even after having completed twenty-one years, must be reported unfit for military service before he is discharged and pensioned, it may naturally be supposed that a considerable amount of feigned disease is to be met with among these men. They are perhaps the most difficult cases with which the army surgeon has to deal; because, in many instances, the disease is not altogether simulated, but a slight affection is exaggerated, and the medical officer is called upon to decide, not whether it really exists, but whether it does so to such an extent as to disqualify the individual for the duties of his profession.

The causes, then, which give rise to the feigning of disease in the army may be divided into four great classes: 1. A desire to avoid entering the army or to be freed from it before the person has become habituated to its customs.* 2. A wish to escape some particular duty or punishment, or to get removed from some station abroad. 3. To escape from the irksome duties of a military life, with which the soldier has become disgusted. 4. To procure his discharge from the service, after having acquired a claim to pension. It devolves upon regimental medical officers, with the commanding officer's concurrence, to recommend soldiers to be discharged on account of disabilities, and it is obviously their duty to be careful not to bring forward any but such as are really unfit; for otherwise, at the same time that a useful man is lost, by unduly increasing the numbers on the pension-list the means of rewarding the really deserving soldiers are diminished, and occasion given for an outcry against the "burden" thus imposed on the country. The soldiers thus recommended are examined by a staff-surgeon, and any whom he deems fit for further service are ordered to return to their duty: they seldom, however, afterwards prove efficient, as they can always, when found fault with, fall back upon "the doctor's" opinion of their unfitness. This, therefore, should prove an additional reason for great circumspection in the performance of this important duty.

In all cases of malingering there is need of great patience and much professional skill on the part of the surgeon to enable him satisfactorily to investigate and decide upon the nature of the alleged disability. "The wiles of soldiers in hospital," says Dr. Cheyne, "will be with more certainty discovered by those who have an accurate knowledge of disease obtained from clinical observation and pathological writings of authority, than by those possessing natural sagacity, in the highest degree, if unassisted by a habit of carefully contemplating and studying disease." But when the surgeon has satisfied himself that the disease is feigned, his work may be said to be only begun: the most difficult part remains, namely, to induce the man to return to his duty. Marshall says, on this subject, "It is only after some experience that a medical officer is aware of the difficulties he has to encounter in his endeavours to reform persevering malingerers. Let him be ever so assiduous, and adopt the most judicious measures for the recovery of simulators who suffer under some

* This cause is in much more active operation in armies raised and maintained by conscription than in those recruited by voluntary enlistment.

real, although only trifling, cause of inability, he will frequently find his measures rendered nugatory by their unwillingness to be restored to the ranks, and the pains they take to retard their convalescence."

Impressed with the importance of the study of this subject, Sir George Ballinghall, in 1835, offered a prize to the pupils attending the class of military surgery in the University of Edinburgh, for the best essay " on the best classification of the feigned and factitious diseases of soldiers and seamen."

"The object which the Professor more immediately had in view, was the formation of such a classification as would enable the surgeon, by it alone, to form an idea, not only of the frequency and success of imposition in any particular disease, but also of adjudging to soldiers who were discharged with a pension, the rate of that pension according as the disease on account of which they were discharged was or was not capable of simulation; premising that, though such a rule might (and probably would) be attended with individual injustice, yet its practical advantages would counterbalance such a minor grievance; because there would be a fixed scale for the amount of pension to be awarded to the soldiers discharged on account of a particular disability, and a great consequent reduction of the pension-list." (Gavin, Preface.)

We have given this quotation in our author's words, because we are by no means sure that we completely understand what the learned Professor wished; but we feel certain that if we have rightly conceived the object he had in view, it has not been attained by Dr. Gavin's work, which gained the prize. Before proceeding to examine the classification adopted in it, however, we must protest against the doctrine implied in the preceding paragraph,—that, for the sake of obtaining a uniformity in the rates of pension awarded for particular disabilities, and a great consequent reduction of the pension-list, we are entitled to disregard the *just* claims of the soldier. We regret deeply that an officer of Sir George Ballinghall's standing, and of such deservedly high reputation, should for a moment have advocated the doctrine of awarding pensions according to an artificial and arbitrary arrangement, without any reference to the merits of the case or the character of the individual, and even in total disregard of the *rights* of those who, in the service of their country, have perilled their lives, and returned to their native lands shattered in constitution, and often unable to earn a livelihood by their own labour.

In the work before us Dr. Gavin states:

" Such a classification as that desired by the Regius Professor—viz. that by it we should be enabled to proportion the rate of pension to be given to soldiers who are to be discharged on account of disease—cannot be obtained by following the arrangement of any nosologist, or the divisions of any of the authors who have treated more particularly of feigned diseases..........An artificial arrangement must therefore be adopted for the occasion; and it appears that that which will conduce most to the stability of such a classification will be an accurate analysis of the symptoms of each disease, as it generally occurs, and a division of those symptoms into *such as are referrible to the feelings of the patient,* and for which the physician must trust nearly *in toto* to his patient, and into such as are *cognizable by the senses or acquired knowledge* of the physician." (p. 47.)

Now, if the existence of disease or disability were evidenced by one symptom alone, this principle of classification might be adopted; but how very seldom is this the case? In almost every instance—external injuries excepted—we must depend partly on the individual's statement, and partly on our own observation; and it is by the harmony or discrepancy existing between these we are led to form conclusions as to the reality and extent of the disease. But suppose it possible to refer every case to one or other of these classes, would this form a just basis for settling the amount of pension; or, because a disease is easily simulated, would it be equitable to assign a low rate in every instance? Most assuredly not: every case ought to be decided on its own merits, without reference to any arbitrary classification of diseases.

Let us take an example of the mode in which this proposed system would work. "Palsy," says Marshall, "is easily and frequently simulated; and, in many instances, considerable difficulty exists in determining whether an alleged disability of this kind is real or feigned." This, therefore, is a disease for which a small pension only should be granted; but we feel sure no one will support this opinion who has witnessed the deplorable cases of paralysis that occasionally occur, in which the individual is reduced to a state of mere vegetative existence. We have, it is true, selected an extreme instance; but the same principle holds good as regards other diseases—that it would be most unjust to award a trifling amount to a man labouring under a *real* disability, because it happened, unfortunately for him, to be one that was easily or frequently simulated with success. It may also be observed, that *time* is an element of considerable importance in deciding upon the claims of a soldier to pension. A disability which, in the early part of his career, would not justify his discharge, might, at a later period, disqualify him for military service, and justly entitle him to a pension; but, under the proposed system, no cognizance could be taken of this circumstance. The object sought for in this classification, then, appears to be either unattainable, or, if attainable, to be a measure fraught with great injustice.

Let us now briefly examine the arrangement adopted in the other two works whose titles are prefixed, and which are the productions of officers who have spent the greater part of their lives among soldiers, and whose attention has been long and successfully directed to this branch of their duty. Their object has been to group the diseases in such a manner as to facilitate the study, by rendering them more easily understood and retained in the memory; and, with this view, they have classed them physiologically.

Dr. Fallot has divided them into three great classes, as they affect the functions of Relation, of Nutrition, and of Reproduction. The first of these is subdivided into, 1st, the internal sensitive apparatus, comprehending the affections which Mr. Marshall has arranged under the head of diseases and lesions of the nervous system; 2d, the external sensitive apparatus which includes the organs of vision, of hearing, of smell, and of touch; 3d, the organs of sound; 4th, the organs of locomotion. In the second class, namely, the functions of nutrition, are comprised affections of, 1st, the organs of respiration; 2d, those of circulation; 3d, those of digestion; 4th, the urinary organs; and, 5th, the organs of assimilation. The third class comprehends the diseases and lesions of the organs of generation.

The classification adopted by Mr. Marshall is similar in its principles, but the details are somewhat differently arranged, and he has added a most important class, under the title of moral disabilities. This similarity affords a striking example of two persons being led to adopt precisely similar views as the result of extended practical observation, because it appears that M. Fallot was not in possession of Marshall's work until he had made very considerable progress in his own. (Preface, p. 9.)

It is not our intention to enter upon a consideration of the numerous expedients adopted by malingerers to attain their object, nor of the ingenious devices occasionally resorted to for the purpose of detecting the imposition, and causing them to return to their duty. Even those who are not likely to be called upon to carry into practice the principles laid down in these works, will find much amusement, and can scarcely fail to derive benefit also, from their perusal. The following may be taken as a fair specimen of Marshall's mode of illustrating his subject. In treating of crooked back, he says:

"Private J. W., — regiment, was sent home from India to be invalided; and on the 10th of June, 1828, was admitted into Fort Pitt General Hospital. He stooped so much that the upper half of his body formed nearly a right angle with his inferior extremities; and he usually moved from place to place with the help of a stick, about two feet long, which he grasped by the middle. This disability was eventually presumed to be feigned, and he was consequently placed on his back on the floor, under the superintendence of a medical officer, when he held his legs in nearly a vertical direction, and complained most piteously upon any attempt being made to put his inferior extremities in the same line with his body. This position was reversed, and for some time he supported himself upon his head, hands, and feet. He soon became tired of this state of prostration: and at last, when he could endure it no longer, he stretched his legs fully out, and lay flat upon his chest and stomach. He then warmly expressed his gratitude to the medical officer for having so effectually cured him of a disability under which he had long suffered; and observed, that if the surgeon of the corps to which he belonged had done as much for him in India, what a happy man he should have been! He was discharged to duty on the 7th August, 1828."

We have already observed how extremely difficult and important it is to induce the simulator to return to his duty. When, after careful observation, the surgeon has satisfied himself that the disability does not exist, or is not of such a nature as to disqualify the man for military service, he should take an opportunity of quietly pointing out to the individual the folly of his conduct and of stating his firm determination to prevent him obtaining a discharge by such means. It does not follow that in these cases the man must be immediately sent out of hospital, for it is often advisable, particularly where the disease is not altogether feigned, but is one of exaggeration of an unimportant lesion, to retain him for a short time under treatment, and thus permit him to return to his duty without the stigma of being a suspected malingerer. "A man who has been in hospital for an alleged disability of a doubtful character should invariably be afforded an opportunity of *giving in*, (in other words of apparently recovering,) without taking him much to task for his conduct; or, in the language of the hospital, he should be *let down softly*." (Marshall, p. 92.) There are two rules connected with this portion of the subject which ought invariably to be remembered by the medical officer. The first is never to allow even the most flagrant imposition to cause him to lose command of his temper, or lead him to use harsh or unjustifiable language to his patient. Such a method, says Dr. Cheyne, "may sometimes intimidate a raw soldier, but will only afford a stronger motive to the hardened knave for perseverance; and it may likewise subject the officer to the just displeasure of his military superior." The other rule is never to adopt any treatment but what would be appropriate if the alleged disease really existed. Occasionally violent remedies, as the actual cautery, blisters, &c., have been employed with the view of forcing the malingerer out of the hospital, but such means are rarely successful. Moreover, if the disease should eventually prove to be real, what must be the feelings of the surgeon who has been pursuing such a line of treatment towards his unfortunate patient? On the other hand, if it is feigned, what confidence can the soldier place in the medical officer who could adopt such measures avowedly for the removal of an affection which never existed?

When the medical officer has failed, after due perseverance in these means, to induce the simulator to return to his duty, it is incumbent on him to report the particulars of the case to his commanding officer, detailing the circumstances which lead him to believe the disease to be feigned. By the articles of war, "any soldier who shall be convicted of malingering, feigning, or producing disease or infirmity, absence from hospital, protracting cure, &c., shall be tried for disgraceful conduct, and be liable to the punishments attached to that offence." When a soldier therefore has been reported as a malingerer, the duty of adopting any further measures rests with the commanding officer, who has the power to assemble a court martial for his trial. As the case must depend mainly on the evidence of the medical officer, who is placed in the position both of accuser and chief witness, he ought, before thus reporting an individual, to be well satisfied in his own mind of the accuracy of his opinion, and be prepared to substantiate it by evidence likely to prove satisfactory to the court. To break down in his proof would be not only injurious to the discipline of the regiment, but most detrimental to his own reputation.

Having thus briefly noticed the means to be used in endeavouring to reform malingerers, we would earnestly impress upon young medical officers, and aspirants for military employment, the necessity of confining themselves strictly to such duties as are clearly within their province. It occasionally happens that an assistant-surgeon, on first joining the service, is affected with a species of military monomania, if we may so term it, and seems to think it incumbent on him to labour zealously to support discipline. Now, it is of great importance, medical officers should guard against this mistake. If they confine themselves to their own duties, treating the sick with skill, attention, and kindness, cooperating with the commanding officer, when requested, in any measures he may wish to be adopted with a view to preserve discipline amongst the men in hospital, and endeavouring, by firm, but mild and judicious measures, to prevent malingering, they will assuredly gain the esteem and respect of their brother officers, and may safely content themselves with leaving the discipline of the regiments to the adjutant; while a contrary line of conduct will give rise to much disagreeable feeling, disturb the harmony of the corps, and eventually bring them unpleasantly under the notice of their commanding officers. This interference in military matters by medical officers arises from a very mistaken notion of their powers,—a notion which we are the more anxious to correct, that it has (no doubt inadvertently,) on one occasion at least, been sanctioned by very high authority. Sir G. Ballinghall, in speaking of corporal punishments, (Outlines of Military Surgery,) cautions military surgeons "against any untimely or undue interference with the discipline of the service, or any vain parade of authority, in the *only case in which their authority can be considered as at all paramount to that of the commanding officer.*" Now, by the regulations of the army, although the medical officers are granted a relative military rank for certain purposes, it is expressly stated that such relative rank is not to extend "to the exercise of any military authority or command whatever." In the case in question therefore, so far from having an authority "paramount to that of the commanding officer," they have no more right to *order* the punishment to be stopped, than the smallest drum-boy in the regiment; their duty is simply to report to the commanding officer when they consider the culprit unable to bear any further punishment without risk of life, but beyond making this report, they have no power to interfere.

The following remark of the Duke of Wellington, though not specially addressed to medical officers, merits well their consideration: "Abstinence from interference in which those who interfere have no authority, and in which they are not called upon to interfere by duty, will save the officers in command or authority abroad, and those placed over them at home, a great deal of unnecessary trouble."

In the preceding pages we have spoken unfavorably of the classification of diseases proposed by Dr. Gavin. We entertain, however, a different opinion of the general merits of his book, which reflects much credit on his talents and industry. It contains an excellent compilation of all that has been written on the subject, and we can confidently recommend it as a work of reference. It is not, however, so well suited for the study of persons unacquainted with the subject, because, like many compilations, it states the opinions of all the various authors without drawing definite conclusions as to their respective value or deducing practical conclusions. The other two works, on the contrary, are eminently practical, and ought to form part of the library of every army surgeon, and be the frequent subject of study of every candidate for admission into the public service.

---

